# A new aging measure captures morbidity and mortality risk across diverse subpopulations from NHANES IV: A cohort study

**DOI:** 10.1371/journal.pmed.1002718

**Published:** 2018-12-31

**Authors:** Zuyun Liu, Pei-Lun Kuo, Steve Horvath, Eileen Crimmins, Luigi Ferrucci, Morgan Levine

**Affiliations:** 1 Department of Pathology, Yale School of Medicine, New Haven, Connecticut, United States of America; 2 Longitudinal Studies Section, Translational Gerontology Branch, National Institute on Aging, National Institutes of Health, Baltimore, Maryland, United States of America; 3 Department of Epidemiology, Johns Hopkins Bloomberg School of Public Health, Baltimore, Maryland, United States of America; 4 Department of Biostatistics, Johns Hopkins Bloomberg School of Public Health, Baltimore, Maryland, United States of America; 5 Department of Human Genetics, David Geffen School of Medicine, University of California Los Angeles, Los Angeles, California, United States of America; 6 Department of Biostatistics, Fielding School of Public Health, University of California Los Angeles, Los Angeles, California, United States of America; 7 Leonard Davis School of Gerontology, University of Southern California, Los Angeles, California, United States of America; 8 Department of Epidemiology, Yale School of Public Health, New Haven, Connecticut, United States of America; Stanford University, UNITED STATES

## Abstract

**Background:**

A person’s rate of aging has important implications for his/her risk of death and disease; thus, quantifying aging using observable characteristics has important applications for clinical, basic, and observational research. Based on routine clinical chemistry biomarkers, we previously developed a novel aging measure, Phenotypic Age, representing the expected age within the population that corresponds to a person’s estimated mortality risk. The aim of this study was to assess its applicability for differentiating risk for a variety of health outcomes within diverse subpopulations that include healthy and unhealthy groups, distinct age groups, and persons with various race/ethnic, socioeconomic, and health behavior characteristics.

**Methods and findings:**

Phenotypic Age was calculated based on a linear combination of chronological age and 9 multi-system clinical chemistry biomarkers in accordance with our previously established method. We also estimated Phenotypic Age Acceleration (PhenoAgeAccel), which represents Phenotypic Age after accounting for chronological age (i.e., whether a person appears older [positive value] or younger [negative value] than expected, physiologically). All analyses were conducted using NHANES IV (1999–2010, an independent sample from that originally used to develop the measure). Our analytic sample consisted of 11,432 adults aged 20–84 years and 185 oldest-old adults top-coded at age 85 years. We observed a total of 1,012 deaths, ascertained over 12.6 years of follow-up (based on National Death Index data through December 31, 2011). Proportional hazard models and receiver operating characteristic curves were used to evaluate all-cause and cause-specific mortality predictions. Overall, participants with more diseases had older Phenotypic Age. For instance, among young adults, those with 1 disease were 0.2 years older phenotypically than disease-free persons, and those with 2 or 3 diseases were about 0.6 years older phenotypically. After adjusting for chronological age and sex, Phenotypic Age was significantly associated with all-cause mortality and cause-specific mortality (with the exception of cerebrovascular disease mortality). Results for all-cause mortality were robust to stratifications by age, race/ethnicity, education, disease count, and health behaviors. Further, Phenotypic Age was associated with mortality among seemingly healthy participants—defined as those who reported being disease-free and who had normal BMI—as well as among oldest-old adults, even after adjustment for disease prevalence. The main limitation of this study was the lack of longitudinal data on Phenotypic Age and disease incidence.

**Conclusions:**

In a nationally representative US adult population, Phenotypic Age was associated with mortality even after adjusting for chronological age. Overall, this association was robust across different stratifications, particularly by age, disease count, health behaviors, and cause of death. We also observed a strong association between Phenotypic Age and the disease count an individual had. These findings suggest that this new aging measure may serve as a useful tool to facilitate identification of at-risk individuals and evaluation of the efficacy of interventions, and may also facilitate investigation into potential biological mechanisms of aging. Nevertheless, further evaluation in other cohorts is needed.

## Abstract 2

Morgan Levine and colleagues describe a phenotypic aging model which can predict health outcomes in individuals from different populations taking into account age, health, chronological age and behaviours.

## Author summary

**Why was this study done?:**

- Aging is one of the leading risk factors for most chronic diseases; therefore, measuring aging has important applications for clinical, basic, and observational research.
- Persons of the same chronological age may vary in their rate of aging, suggesting that chronological age is an imperfect proxy of biological aging.
- Based on traditional clinical chemistry biomarkers, we recently developed a novel aging measure, Phenotypic Age, which can differentiate mortality risk among persons at the same chronological age.
- However, little is known about the applicability of this new aging measure for differentiating morbidity and mortality risk across diverse subpopulations such as healthy and unhealthy groups, distinct age groups, and persons with various race/ethnic, socioeconomic, and health behavior characteristics.

**What did the researchers do and find?:**

- We calculated Phenotypic Age for 11,432 adults aged 20–84 years and 185 oldest-old adults top-coded at age 85 years from NHANES IV and assessed its association with morbidity and mortality.
- We found that, overall, Phenotypic Age was highly predictive of mortality even after adjusting for chronological age. This mortality prediction was valid across different subpopulations, stratified by age, race/ethnicity, education, disease count, health behaviors, and cause of death. We also observed a strong association between Phenotypic Age and the disease count a person had, after adjusting for chronological age.

**What do these findings mean?:**

- Phenotypic Age can facilitate identification of at-risk individuals for a number of diverse conditions and causes of death. Further, it captures risk stratification in both the healthiest and the unhealthiest populations.
- In clinical research, it may serve as a useful tool for evaluating intervention efficacy, avoiding the need for decades of follow-up.
- Phenotypic Age may also be applicable to basic and observational research, shedding light on factors that alter the pace of aging, and facilitating investigation into potential biological mechanisms and environmental stressors.

## Introduction

Rapid population aging represents a major public health burden, as aging is one of the leading risk factors for most major chronic diseases [1,2]. As a result, preventive strategies and interventions that promote healthy aging are critical. While everyone ages, the rate at which aging occurs is heterogeneous, and between-person variations in the pace of aging manifest as differences in susceptibility to death and disease. Thus, differentiating aging in individuals of the same chronological age, particularly in early life, will facilitate secondary and tertiary prevention through earlier identification of high-risk individuals or groups. However, a key issue remains in how to measure aging. Further, to be applicable to the clinical setting, such assessment should be easy to conduct using existing instruments, must do a better job at capturing risk stratification than current tools, and should be able to differentiate risk prior to manifestation of disease or disability.

One method for determining whether a person appears younger or older than expected on a biological or physiological level is to compare observable characteristics, reflecting functioning or state, to the characteristics observed in the general population for a given chronological age. A number of aging measures have been proposed using molecular variables, the most prominent being epigenetic clocks (expressed as DNA methylation age, in units of years) [3] and leukocyte telomere length [4]. We and others have previously shown that while these measures are phenomenal age predictors—especially DNA methylation age—their associations with aging outcomes above and beyond what is explained by chronological age is weak to moderate [5–11]. Conversely, aging measures based on clinically observable data, or phenotypes, tend to be more robust predictors of aging outcomes [12–15]. The differences in prediction between these 2 types of measures could reflect that molecular measures may only capture 1 or a small number of changes involved in the multifactorial aging process, while on the other hand, clinical measures may represent the manifestations of multiple hallmarks of aging occurring at the cellular and intracellular levels [12,13,15–18]. While composite scores based on traditional clinical chemistry measures are not mechanistic, their better performance and relative affordability and practicality compared to current molecular measures may make them more suitable for evaluating the effects of aging interventions on an organismal scale, and/or identifying groups at higher risk of death and disease.

Among the existing clinical measures, the majority were generated based on associations between composite variables and chronological age—with no integration of information on how the variables influence morbidity and mortality. Given that individuals vary in their rate of aging, chronological time is an imperfect proxy for building an aging measure [19]. Recently, we developed a new metric, Phenotypic Age (in units of years), that incorporates composite clinical chemistry biomarkers based on parametrization from a Gompertz mortality model [12]. Rather than predicting chronological age—as previous measures have done—this measure is optimized to differentiate mortality risk among persons of the same chronological age, using data from a variety of multi-system clinical chemistry biomarkers. In general, a person’s Phenotypic Age signifies the age within the general population that corresponds with that person’s mortality risk. For example, 2 individuals may be 50 years old chronologically, but one may have a Phenotypic Age of 55 years, indicating that he/she has the average mortality risk of someone who is 55 years old chronologically, whereas the other may have a Phenotypic Age of 45 years, indicating that he/she has the average mortality risk of someone who is 45 years old chronologically.

The goal of this study was to evaluate the applicability of this measure by (1) assessing whether it is a robust predictor of all-cause mortality compared to traditional risk factors, (2) establishing how it relates to various causes of death and/or comorbid conditions, and (3) determining generalizability through assessing whether this new measure is predictive of long-term mortality risk in a variety of subpopulations, e.g., various age groups, racial/ethnic groups, persons with various socioeconomic status (SES), persons with various smoking/drinking habits, disease-free individuals, and groups with various disease counts.

## Methods

### Study population

We previously developed Phenotypic Age using data from NHANES III (the third National Health and Nutrition Examination Survey) (1988–1994) [12]. The independent validation sample used here was from NHANES IV (1999–2010, *n* = 14,008). We excluded participants with missing data on biomarkers or who did not complete at least 8 hours of fasting prior to blood sampling (*n* = 1,368), with missing data on follow-up time (*n* = 15), or who did not have survey weights (*n* = 1,008). The final analytic sample included *n* = 11,432 adults aged 20–84 years (S1 Table) and 185 oldest-old adults top-coded at age 85 years. On average, the persons excluded tended to be older (2.5 years on average) and were 40% more likely to self-identify as non-Hispanic black. Details of recruitment, procedures, population characteristics, and study design for NHANES are provided through the Centers for Disease Control and Prevention [20] (https://www.cdc.gov/nchs/nhanes/index.htm). Briefly, NHANES is an ongoing program by the National Center for Health Statistics involving a series of independent, nationally representative cross-sectional surveys designed to assess the health and nutritional status of adults and children in the US. It began in the early 1960s focusing on different population groups and health topics and became a continuous program that has had a changing focus on a variety of health and nutrition measurements to meet emerging needs since 1999. Using both at-home interviews and examinations performed at a mobile examination center, NHANES collects a wide range of information (e.g., via demographic, socioeconomic, dietary, and health-related questions, and medical and physiological measurements) from a nationally representative sample each year in counties across the country [20]. NHANES is approved by the National Center for Health Statistics Research Ethics Review Board, and all participants provide informed consent. Data used in this study are de-identified and publicly available (https://www.cdc.gov/nchs/nhanes/index.htm). This study received approval from the Yale Human Investigation Committee on 15 November 2017 following an expedited review.

### Mortality

Mortality follow-up was based on linked data from records taken from the National Death Index through December 31, 2011, provided through the Centers for Disease Control and Prevention [20]. Data on mortality status and length of follow-up (in person-months) were available for nearly all participants (*n* = 15 with missing data on follow-up time). Out of 9 underlying causes of death and an “other” category that were provided in the linked data, 7 were used to assess cause-specific mortality in our study—heart disease, cancer, chronic lower respiratory disease, cerebrovascular disease, diabetes, influenza or pneumonia, and nephritis/nephrosis. Alzheimer disease was not considered in the cause-specific analysis due to the small number of deaths assigned to this cause. Accidents were not assessed due to the fact that many may not be age-related, and it is impossible to differentiate age- versus non-age-related accidental death.

### Phenotypic Age

We calculated Phenotypic Age in accordance with the method described previously [12]. Briefly, Phenotypic Age is calculated using chronological age and 9 biomarkers (albumin, creatinine, glucose, [log] C-reactive protein [CRP], lymphocyte percent, mean cell volume, red blood cell distribution width, alkaline phosphatase, and white blood cell count) that were selected using a Cox proportional hazard elastic net model for mortality based on 10-fold cross-validation. The algorithm for calculating Phenotypic Age is based on parametrization of 2 Gompertz proportional hazard models—one fit using all 10 selected variables, and the other fit using only chronological age. The resulting final equation for calculating Phenotypic Age is as follows:

$$\text{Phenotypic}\;\text{Age}=141.50+\frac{\text{ln}\left[-0.00553\times \text{ln}\left(1-\text{xb}\right)\right]}{0.09165}$$

where

xb=−19.907−0.0336×albumin+0.0095×creatinine+0.0195×glucose+0.0954×ln(CRP)−0.0120×lymphocytepercent+0.0268×meancellvolume+0.3356×redbloodcelldistributionwidth+0.00188×alkalinephosphatase+0.0554×whitebloodcellcount+0.0804×chronologicalage

Finally, we calculated a measure, Phenotypic Age Acceleration (PhenoAgeAccel), defined as the residual resulting from a linear model when regressing Phenotypic Age on chronological age. Therefore, PhenoAgeAccel represents Phenotypic Age after accounting for chronological age (i.e., whether a person appears older [positive value] or younger [negative value] than expected, physiologically, based on his/her age).

### Health and demographic characteristics

Age categories, race/ethnicity, education, body mass index (BMI), disease count, smoking status, and drinking habits were considered for stratified analyses. Four age categories (20–39, 40–64, 65–84, and 85+ years) and 3 racial/ethnic groups (non-Hispanic white, non-Hispanic black, and Hispanic) were considered. Note that for presenting Kaplan–Meier curves, we used a different set of 4 age categories (20–49, 50–64, 65–74, and 75–84 years) to demonstrate the robustness of the results. A 4-category education variable was used to approximate SES. Categories included less than high school (HS), HS/general educational development (GED), some college (having attended college but not receiving at least a bachelor’s degree), or college (having a bachelor’s degree or higher). BMI was calculated as weight in kilograms divided by height in meters squared. Underweight was defined as BMI < 18.5 kg/m^2^, normal was defined as 18.5 ≤ BMI < 25.0 kg/m^2^, overweight was defined as 25.0 ≤ BMI < 30.0 kg/m^2^, and obese was defined as BMI ≥ 30 kg/m^2^. Chronic diseases included 10 coexisting self-reported conditions: congestive heart failure, stroke, cancer, chronic bronchitis, emphysema, cataracts, arthritis, type 2 diabetes, hypertension, and myocardial infarction. Based on the disease counts, we created a variable with 5 categories—no disease, 1 disease, 2 diseases, 3 diseases, and 4 or more diseases (with the last two categories combined in subgroup analyses). Three smoking status categories were created, which included never smokers (<100 cigarettes during one’s lifetime), former smokers (100 or more cigarettes during one’s lifetime, but not actively smoking during recent time frame), and current smokers (ongoing smoking habit). Two drinking variables were created—a binary binge drinking indicator (in which binge drinking was defined as having 5+ alcoholic beverages at a time at least once per month) and a 6-category alcohol intake variable (never, none in past year, <1 drink per month, 1–3 drinks per month, 1–3 drinks per week, 4+ drinks per week). All the information was collected through a questionnaire or physical examination at the time of survey.

In this study, when comparing the predictive performance of Phenotypic Age with that of traditional risk factors, we not only considered the individual biomarkers that were already included in Phenotypic Age, but also considered disease count, BMI, total cholesterol, and systolic blood pressure, given that they are commonly considered risk factors for death and disease in both observational studies and clinical practice [21–25]. Data on total cholesterol were obtained from blood analyses, and data on systolic blood pressure were obtained from examination at the time of survey.

### Statistical analyses

**Fig 1:**
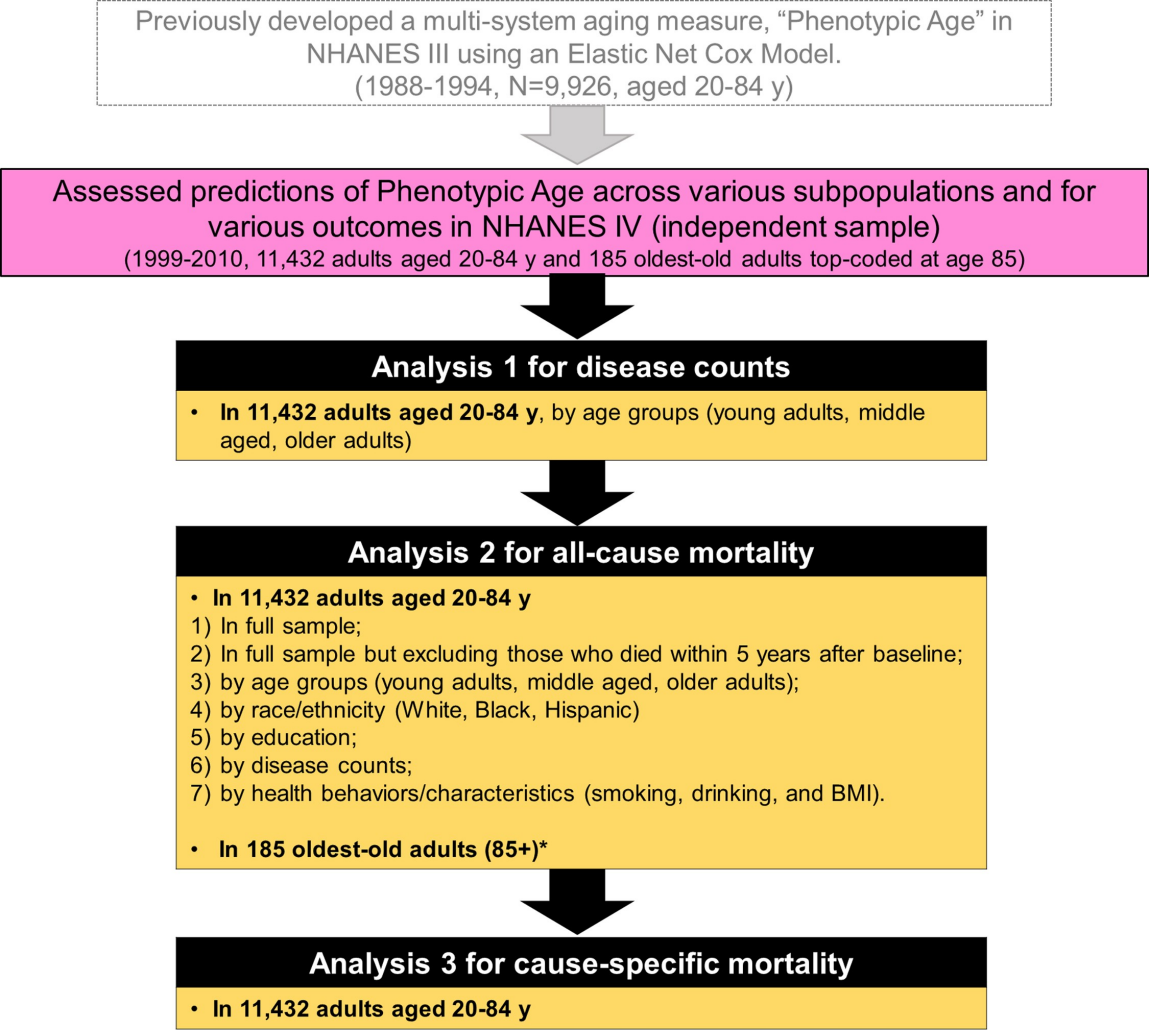
The analytic plan for this study. NHANES III and IV refer to the third and fourth National Health and Nutrition Examination Survey. *We adjusted for chronological age and sex in all models except those in the oldest-old adults. As mentioned in the Methods, we ran 2 parametric proportional hazard models (Gompertz distribution) in this age group, one unadjusted and another with adjustment for disease count, rather than chronological age (unknown).BMI, body mass index.

The analytic plan for this study is briefly described in Fig 1. Using data from NHANES IV, age-stratified ordinary least squares regression models were first used to estimate the association between disease count and Phenotypic Age within 3 age categories (20–39 years, 40–64 years, and 65–84 years). Based on these regression equations, we then estimated the incremental increase in PhenoAgeAccel for participants in each of the disease count categories in comparison to participants with no disease.

Next, a parametric proportional hazard model (Gompertz distribution) was used to assess the association between Phenotypic Age and all-cause mortality, with adjustment for chronological age and sex. To further evaluate robustness, age-stratified models and a model that excluded short-term mortality (within 5 years after baseline) were also run to ensure the mortality prediction was not driven by older ages and/or an end-of-life phenotype. Participants were then grouped into quintiles for PhenoAgeAccel, so that the highest quintile represented individuals who were most at risk of death for their age—i.e., those whose Phenotypic Age was the highest relative to their chronological age. We then plotted Kaplan–Meier curves for persons in the highest 20% versus the lowest 20%. We also compared predicted median life expectancy at age 65 years by sex and the 5 quintiles for PhenoAgeAccel. Next, receiver operating characteristic (ROC) curves were used to compare the 10-year mortality risk prediction of Phenotypic Age to predictions based on individuals’ clinical chemistry biomarkers and routine risk assessment tools (e.g., based on systolic blood pressure, the biomarkers, and BMI).

Cause-specific mortality risk as a function of Phenotypic Age was assessed via Fine and Gray’s competing risk models [26]. To determine whether Phenotypic Age could differentiate risk in population subgroups (e.g., healthy participants), we conducted the all-cause mortality analysis again by race/ethnicity, education, disease count, BMI, smoking status, and drinking habits.

Participants aged 85+ years (oldest-old adults) were excluded from all prior analyses given that age was top-coded (i.e., everyone aged 85+ years was coded as being age 85 years) for identity protection; therefore, to test mortality associations in this group, we used 2 parametric proportional hazard models (Gompertz distribution), one adjusted for sex and another with adjustment for sex and disease count, rather than chronological age (unknown).

All analyses were performed using R version 3.4.1 (2017-06-30) and STATA version 14.0 software (StataCorp, College Station, TX).

## Results

The basic characteristics of the study participants are shown in S1 Table. The mean age of the 11,432 adults was 45.5 years, and about half of the sample were women (50.8%). Young (20–39 years) and middle aged (40–64 years) adults accounted for 40% and 45%, respectively. Three-quarters of participants self-identified as non-Hispanic white, about 11% were non-Hispanic black, and 13% were Hispanic. One-quarter of participants had a college degree, about 30% had some college education, one-quarter had a HS education, and about 19% had not graduated from HS or received a GED. Half of the sample were never smokers, while the other half were approximately equal parts former and current smokers. Approximately 15% had binge drinking tendencies over the past year. Finally, proportions of normal BMI, overweight, and obese were each about one-third.

### Prevalence of disease

**Fig 2:**
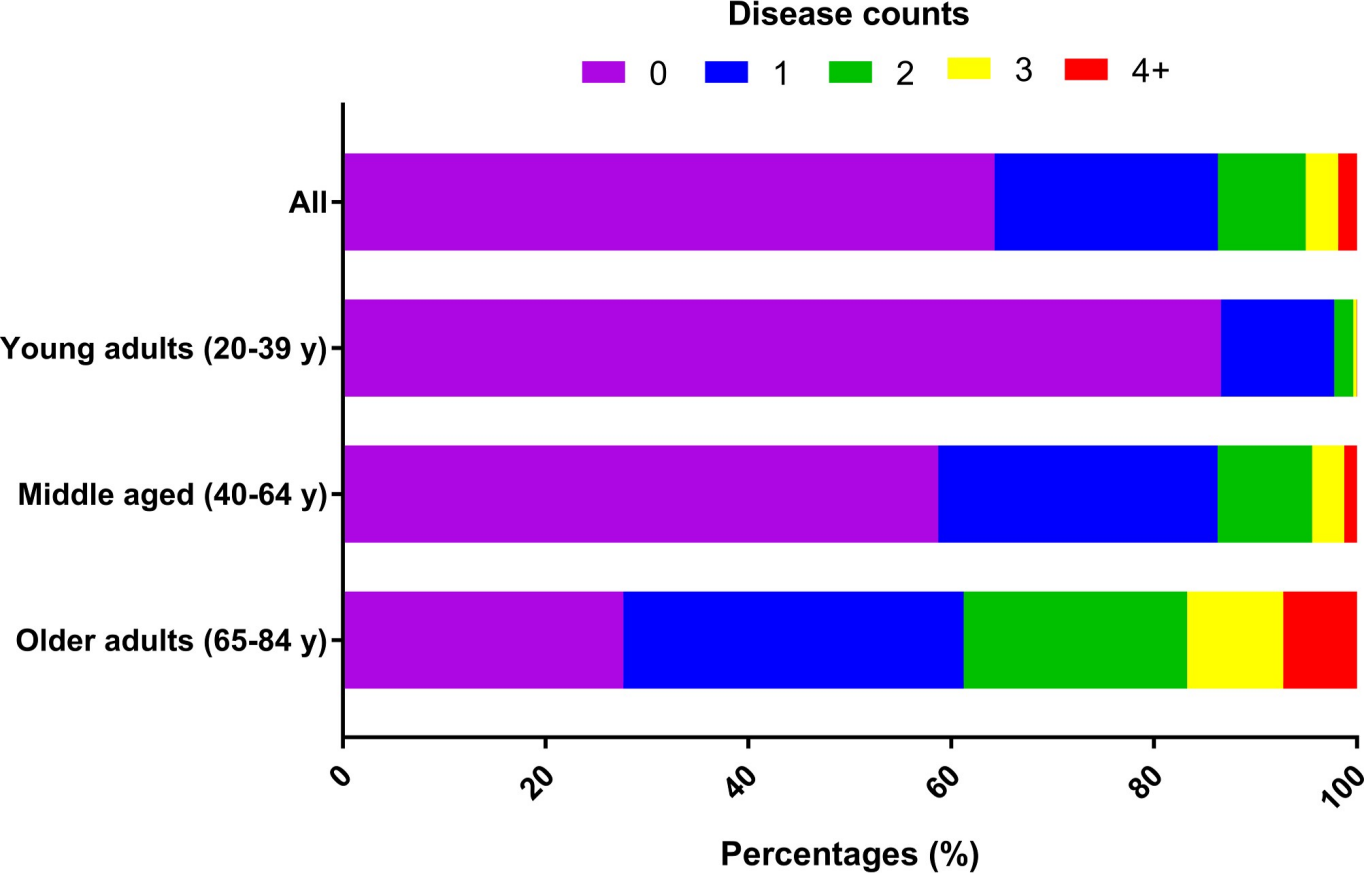
Frequency of disease counts overall and by age category. The *y-*axis depicts the various age groups. The *x-*axis represents the relative proportions of persons in each disease count category (designated by colors).

Fig 2 presents the disease counts overall and by age category. Approximately two-thirds (64%) of the study participants were disease-free at their interview, while 22% reported having been diagnosed with 1 chronic disease, 9% reported 2 diseases, 3% reported 3 diseases, and only 2% reported at least 4 coexisting chronic diseases. As expected, the majority (87%) of young adults (aged 20–39 years) were free of disease, compared to 59% of middle aged (40–64 years) and only a quarter (28%) of older adults. Additionally, 7% of older adults had 4 or more chronic diseases, while only 1% of middle aged adults and essentially no young adults reported 4 or more disease diagnoses.

### PhenoAgeAccel according to disease count and age category

**Fig 3:**
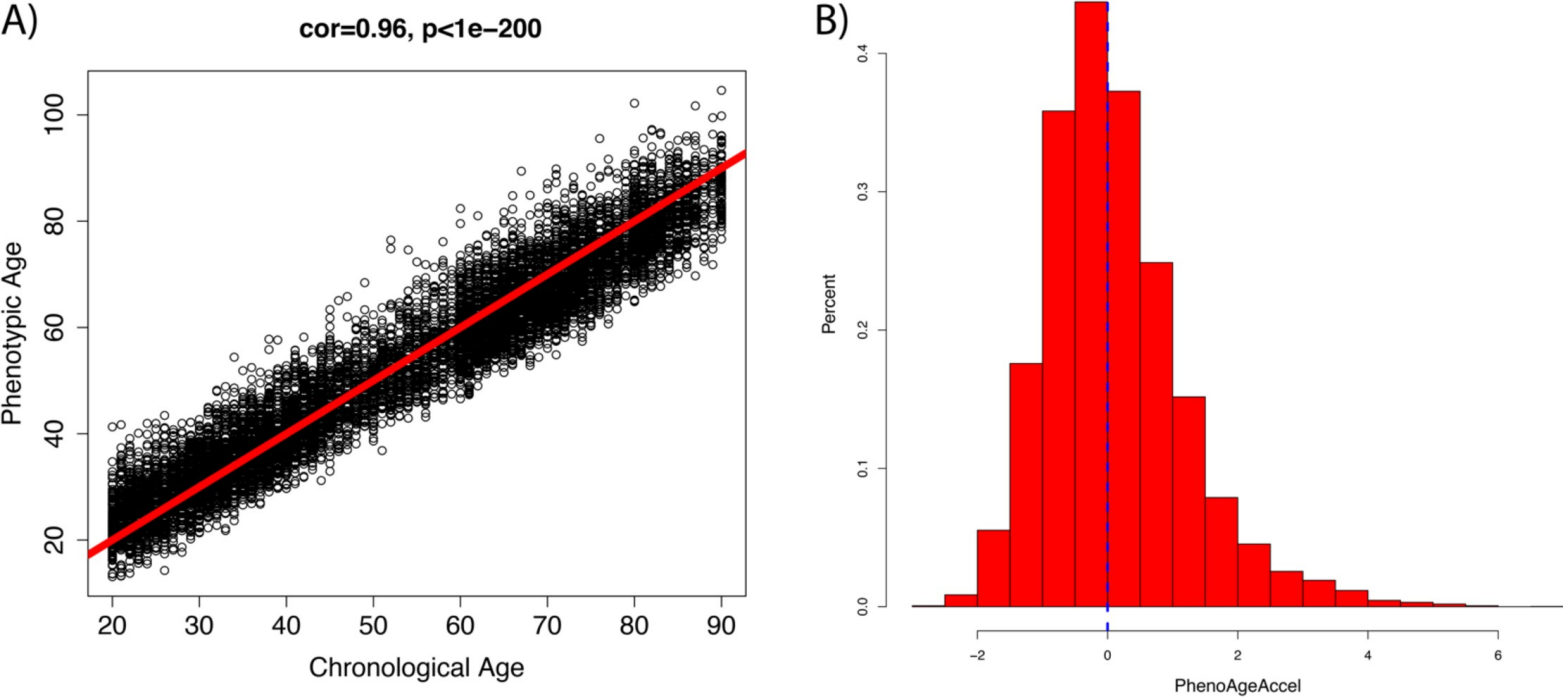
Relationship between Phenotypic Age, chronological age, and PhenoAgeAccel. (A) As expected, Phenotypic Age was highly correlated with chronological age, partially due to the fact that it includes chronological age. The red line depicts the expected Phenotypic Age for each chronological age, with points above the line depicting people who were phenotypically older than expected, and points below the line depicting those who were phenotypically younger than expected. (B) PhenoAgeAccel was fairly normally distributed, with a mean of 0 (blue line), a standard deviation of 1, and a median of −0.13.

Fig 3 shows the correlation between Phenotypic Age and chronological age, as well as the distribution of PhenoAgeAccel—the residual of Phenotypic Age regressed on chronological age. Phenotypic Age and chronological age are highly correlated; part of this is due to the fact that age is in the Phenotypic Age measure. Consistent with many of the previous aging measures, we also observed that the Phenotypic Age of young adults tended to be overestimated, while the Phenotypic Age of older adults tended to be underestimated. Given that the Δ for Phenotypic Age and chronological age would be biased by age, we estimated the residual for Phenotypic Age, referred to as PhenoAgeAccel. A score of 0 suggests a Phenotypic Age that is consistent with what is expected based on an individual’s chronological age, whereas a positive value suggests that the person has clinical chemistry biomarkers that characterize an older person, and a negative value suggests the person has the clinical chemistry profile of a person younger than expected. While the measure is fairly normally distributed, most of the outliers tend to be in the positive (older) direction.

**Fig 4:**
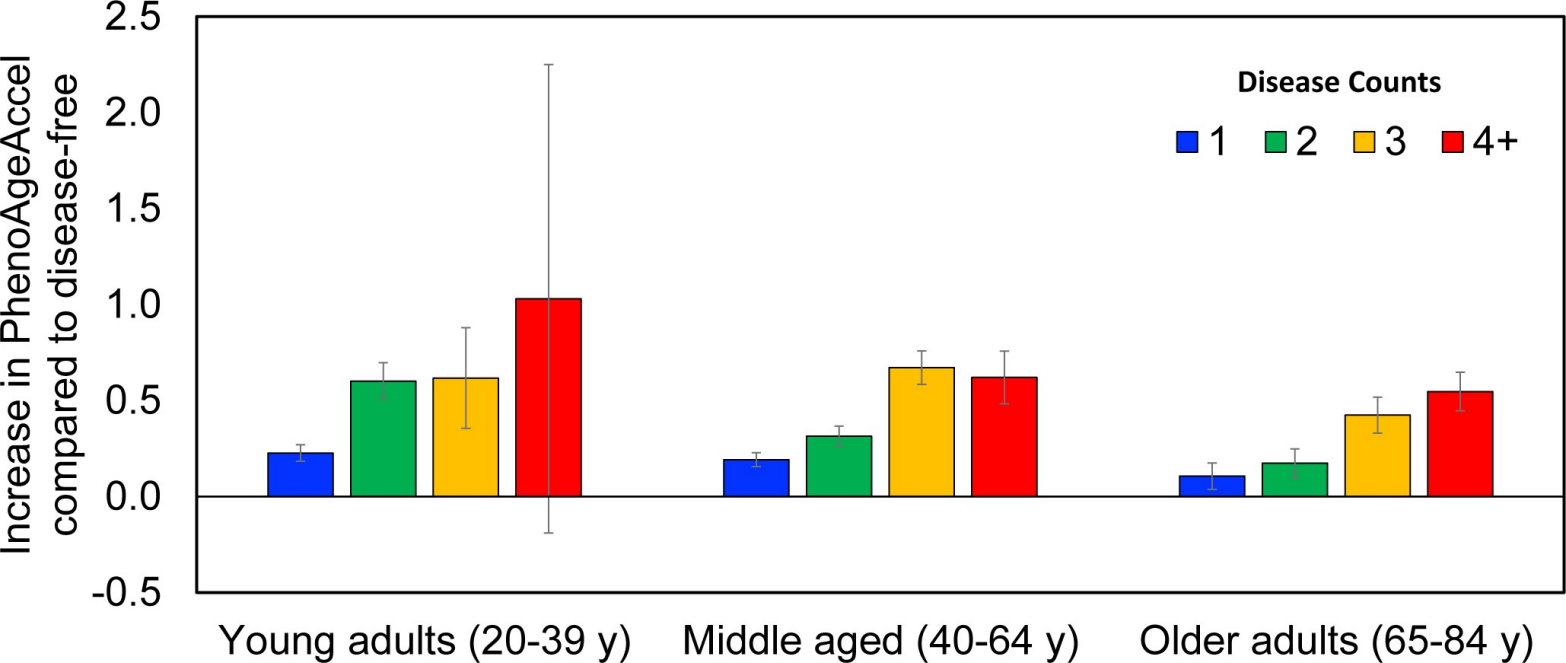
Predicted increase in PhenoAgeAccel for each disease count by age category. The *y-*axis depicts the increase in PhenoAgeAccel compared to persons who were disease-free. The *x-*axis shows groups categorized based on chronological age and the number of diseases each participant had. For all age categories, we observed that PhenoAgeAccel was positive among persons who were diagnosed with 1 or more chronic diseases.

Fig 4 shows predicted increases in PhenoAgeAccel for each disease count category, compared to persons with no diagnosis of disease. Overall, participants with disease had older Phenotypic Age compared to those without disease. For instance, among young adults, those with 1 disease were on average 0.2 years older phenotypically than disease-free persons, and both those with 2 diseases and those with 3 diseases were about 0.6 years older phenotypically. In middle aged adults, compared to those who were disease-free, those with 1 disease had a Phenotypic Age that was on average 0.2 years older, those with 2 diseases had a Phenotypic Age that was 0.3 years older, those with 3 diseases had a Phenotypic Age that was 0.6 years older, and those with 4 or more diseases had a Phenotypic Age that was 0.7 years older. Finally, for older adults, Phenotypic Age increased consistently as a function of disease count, with those reporting 1 disease having a Phenotypic Age that was on average 0.1 years older than disease-free participants, those with 2 diseases having a Phenotypic Age 0.2 years older, those with 3 diseases having a Phenotypic Age 0.4 years older, and those with 4 or more diseases having a Phenotypic Age 0.6 years older.

### Associations of Phenotypic Age with all-cause mortality

Table 1 shows the association between Phenotypic Age and all-cause mortality, based on proportional hazard models with Gompertz distribution. In the full sample, each 1-year increase in Phenotypic Age (after adjusting for chronological age) increased the risk of mortality by 9% (hazard ratio [HR] = 1.09, 95% CI = 1.08–1.10). When restricting the sample to participants who survived at least 5 years after baseline, we found consistent results, such that each 1-year increase in Phenotypic Age was associated with an 8% increase in mortality risk. When examining mortality within age-stratified groups, we found that Phenotypic Age was predictive in all age groups, such that each 1-year increase in Phenotypic Age was associated with a 13% increased mortality risk in young adults, a 10% increase in middle aged adults, and a 8% increase in older adults. Finally, we found that, on average, females were phenotypically younger than males (β = −1.34, *P* < 0.001); therefore, we compared sex-stratified models of all-cause mortality associations and found identical results for both sexes (HR = 1.09, 95% CI = 1.07–1.11).

**Table 1:** Association of Phenotypic Age with all-cause mortality and disease-specific mortality.

Mortality category		Number of deaths	Hazard ratio (95% CI)	*z-*Score	*P* value
**All-cause**	Full sample	871	1.09 (1.08–1.10)	15.03	<0.001
	Those with 5+ years of survival	389	1.08 (1.06–1.10)	7.84	<0.001
	Young adults (20–39 years)	32	1.13 (1.09–1.18)	6.47	<0.001
	Middle aged adults (40–64 years)	247	1.10 (1.08–1.12)	10.29	<0.001
	Older adults (65–84 years)	592	1.08 (1.06–1.09)	10.40	<0.001
**Disease-specific**	Heart disease	141	1.10 (1.07–1.13)	7.38	<0.001
	Cancer	227	1.07 (1.05–1.09)	6.70	<0.001
	Chronic lower respiratory disease	52	1.07 (1.04–1.11)	4.16	<0.001
	Cerebrovascular disease	56	1.03 (0.98–1.09)	1.26	0.208
	Diabetes	26	1.19 (1.13–1.26)	6.64	<0.001
	Influenza or pneumonia	24	1.12 (1.08–1.16)	6.43	<0.001
	Nephritis/nephrosis	15	1.20 (1.16–1.25)	9.67	<0.001

Results are based on parametric survival models (Gompertz distribution). All models were adjusted for chronological age and sex.

**Fig 5:**
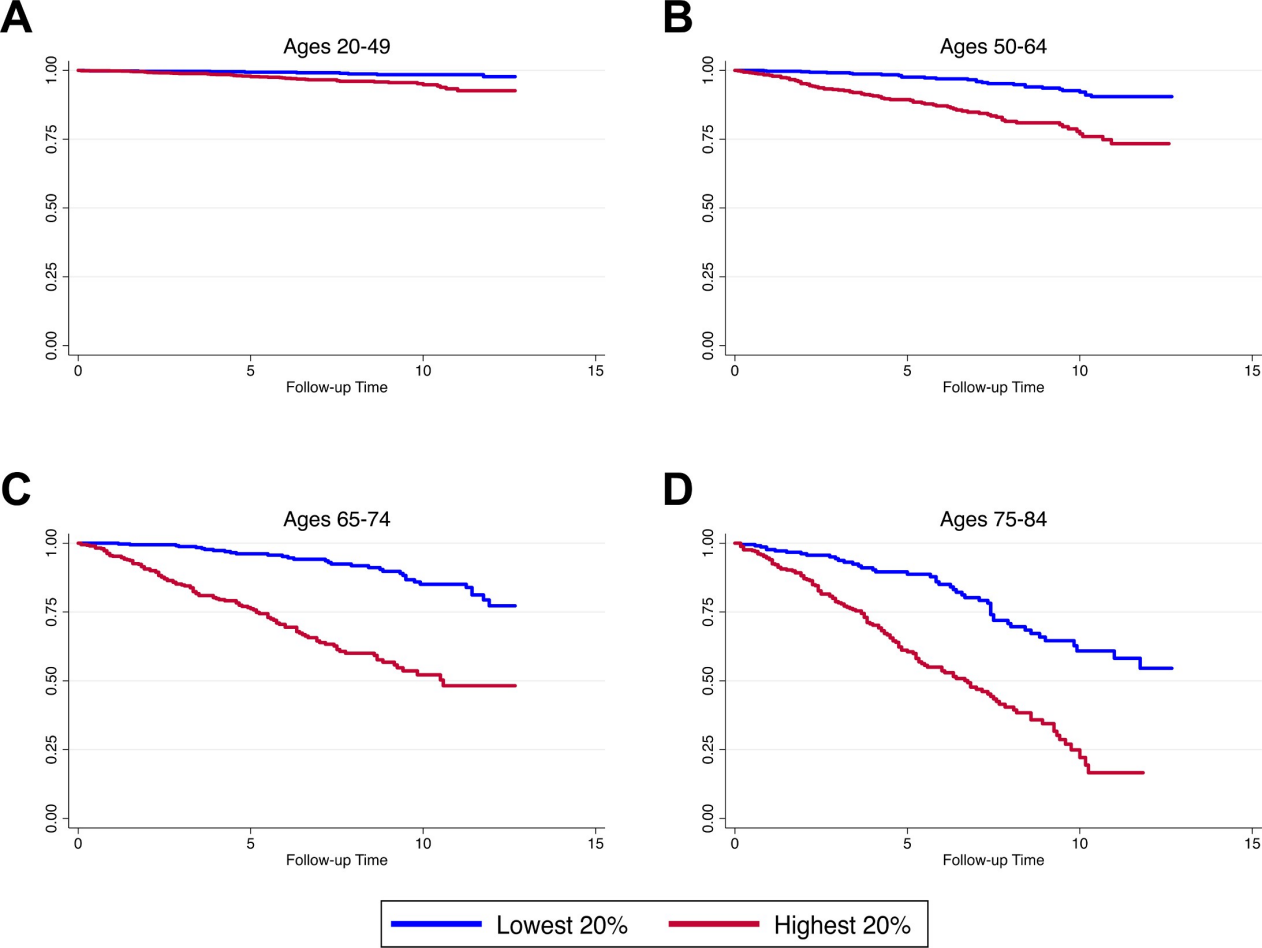
Kaplan–Meier curves for persons in the highest 20% versus the lowest 20% of PhenoAgeAccel. The *y-*axis indicates the survival rate, and the *x-*axis indicates follow-up time (in years).

As shown in Fig 5, we found that those with the highest Phenotypic Ages relative to their chronological ages had much steeper declines in survival over the approximately 12.5 years of follow-up. Interestingly, the high-risk groups (highest 20% of PhenoAgeAccel) appeared to have mortality rates that were similar, or in some cases higher, than those of persons in the low-risk groups (lowest 20% of PhenoAgeAccel) who were 10 years older chronologically. For instance, among persons aged 50–64 years at baseline, about 25% of the high-risk group had died after 10 years of follow-up. Conversely, among persons aged 65–74 years, only about 20% of those in the low-risk group had died after 10 years of follow-up. For persons aged 65–74 years in the high-risk group, about half had died after 10 years, compared to only about 67% of the low-risk group who were aged 75–84 years at baseline.

**Fig 6:**
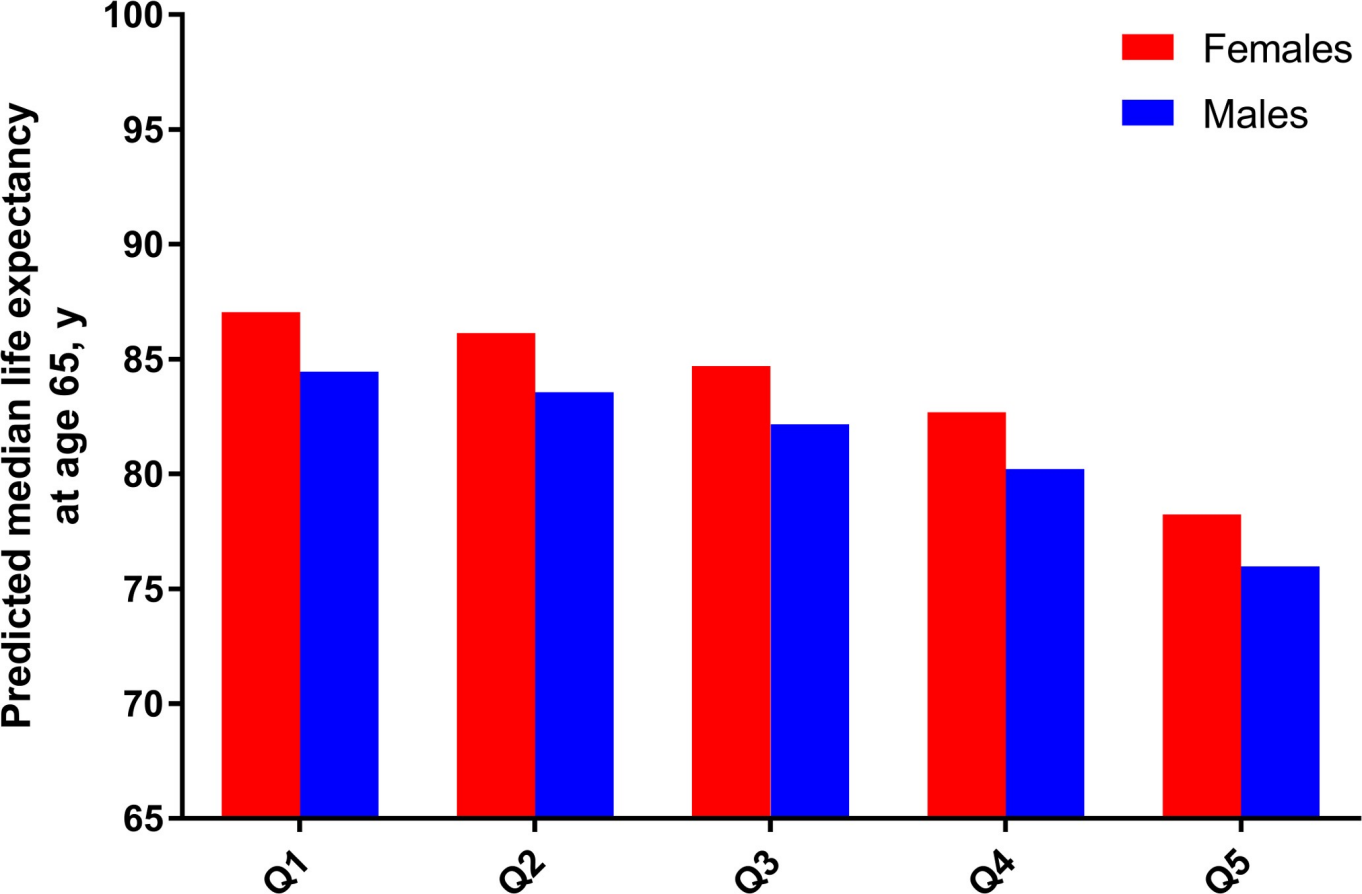
Predicted median life expectancy at age 65 years by sex and the 5 quintiles for PhenoAgeAccel. Q1–Q5 indicate the 5 quintiles of PhenoAgeAccel. Results are based on parametric survival models (Gompertz distribution) that include quintiles of PhenoAgeAccel, chronological age, and sex. Estimates represent the predicted age by which 50% of the population is expected to have died for each sex by quintile group, assuming a baseline age of 65 years.

Fig 6 presents predicted median life expectancy at age 65 years by sex and the 5 quintiles of PhenoAgeAccel. Results showed that 65-year-old females in the lowest quintile (low-risk, or healthiest) had a predicted median life expectancy of about 87 years, while females in the highest quintile (high-risk, or unhealthiest) had a predicted life expectancy of just over 78 years. Similarly, 65-year-old males in the lowest quintile had a predicted median life expectancy of about 85 years, while males in the highest quintile had a predicted life expectancy of just under 76 years.

**Fig 7:**
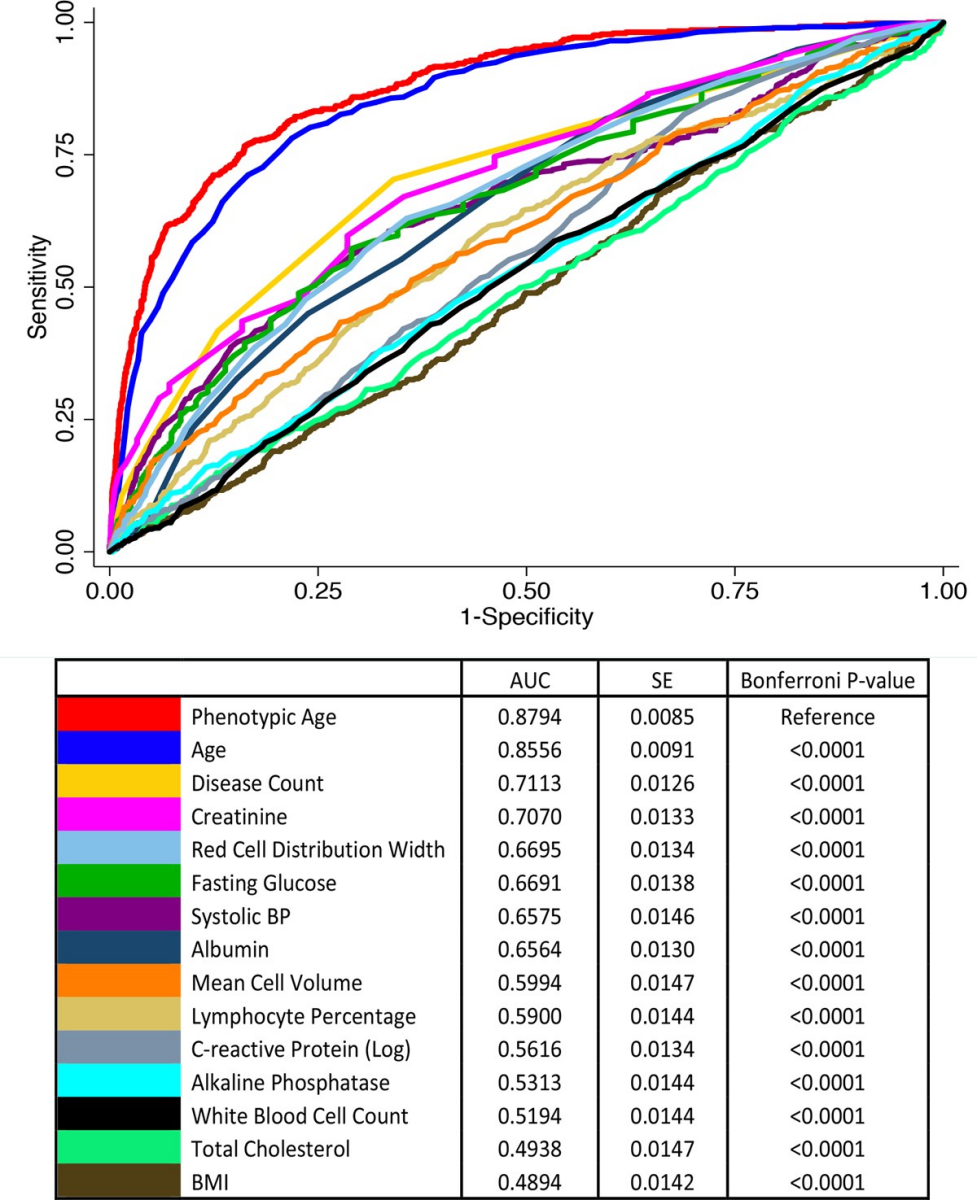
Receiver operating characteristic curves for 10-year mortality. AUC, area under the curve; BMI, body mass index; BP, blood pressure; SE, standard error.

ROC curves (Fig 7) revealed that Phenotypic Age, with an area under the curve (AUC) of 0.88, significantly outperformed the individual clinical chemistry measures and other risk factors. The next highest performing measures were chronological age, with an AUC of 0.86; disease count, with an AUC of 0.71; and serum creatinine, with an AUC of 0.71. Four measures had AUCs between 0.60–0.69 (red blood cell distribution width, fasting glucose, systolic blood pressure, and albumin), 5 had AUCs between 0.50–0.59 (mean cell volume, lymphocyte percentage, CRP, alkaline phosphatase, and white blood cell count), and 2 had AUCs less than 0.50 (total cholesterol and BMI).

As shown in Table 2, we reexamined the ROC curves using various combinations of variables, with and without Phenotypic Age included. We found that in all cases, Phenotypic Age contributed additional predictive power to all models. More interestingly, Phenotypic Age alone was more predictive of 10-year mortality than a model that included chronological age, demographics (race/ethnicity and sex), SES (education), and disease count. It was only when chronological age, demographics, SES, disease count, and health behaviors (smoking, alcohol intake, binge drinking, and BMI) were all included in a single model that the AUC started to approach the AUC for Phenotypic Age alone. Nevertheless, Phenotypic Age still added substantial predictive power when included with those variables, suggesting that it captures something above and beyond what can be explained for mortality risk by demographics, SES, disease, and health behaviors.

**Table 2:** AUC for 10-year mortality for combinations of variables.

Variables included	AUC for 10-year mortality	
	Without Phenotypic Age	With Phenotypic Age
Age	0.855	0.879
Age, demographics, SES	0.867	0.886
Age, demographics, SES, disease count	0.870	0.887
Age, demographics, SES, disease count, and health behaviors	0.878	0.892

The AUC for Phenotypic Age alone was 0.879. Demographic variables included race/ethnicity and sex. SES refers to education. Health behavior variables included smoking, alcohol intake, binge drinking, and BMI categories.

AUC, area under the curve; SES, socioeconomic status.

### Associations of Phenotypic Age with disease-specific mortality

As shown in Table 1, as expected, there were large frequency differences between the disease-specific causes of death, with the numbers of deaths ranging from 15 (nephritis/nephrosis) to 227 (cancer). Nevertheless, although Phenotypic Age was trained to predict all-cause mortality (which was heavily skewed towards cardiovascular and cancer deaths), we found that Phenotypic Age was predictive of disease-specific mortality including heart disease, cancer, chronic lower respiratory disease, diabetes, influenza/pneumonia, and nephritis/nephrosis, with exception of cerebrovascular disease mortality (HR = 1.03, 95% CI = 0.98–1.09). HRs were the highest for diabetes and nephritis/nephrosis, suggesting that a 1-year increase in Phenotypic Age relative to chronological age increases the risks of death from these causes by about 20%. For the other causes (aside from cerebrovascular disease), a 1-year increase in Phenotypic Age increased risk by between 7% (cancer and chronic lower respiratory disease) and 12% (influenza/pneumonia).

### Associations of Phenotypic Age with all-cause mortality in population subgroups

Given the need to identify aging measures that are generalizable across various populations, we examined all-cause mortality associations using stratified models. In general, we found consistent associations regardless of the subgroup (Table 3). Consistent with the HR for the overall population (Table 1) of 1.09, HRs from stratified models ranged from 1.04 (persons with 3+ diseases) to 1.15 (underweight persons). When all variables, such as age, race/ethnicity, sex, education, smoking, and drinking, were adjusted for, Phenotypic Age remained significantly associated with mortality (HR = 1.06, *P* < 0.001).

**Table 3:** Associations between Phenotypic Age and all-cause mortality in population subgroups.

Subgroup	Hazard ratio (95% CI)	*z-*Score	*P* value
**Race/ethnicity**			
Non-Hispanic white	1.09 (1.08–1.11)	11.91	<0.001
Non-Hispanic black	1.07 (1.05–1.10)	6.97	<0.001
Hispanic	1.08 (1.06–1.11)	5.95	<0.001
**Education**			
Less than HS	1.09 (1.07–1.10)	10.10	<0.001
HS/GED	1.09 (1.07–1.11)	7.56	<0.001
Some college	1.08 (1.06–1.10)	7.18	<0.001
College	1.08 (1.04–1.12)	3.93	<0.001
**Smoking**			
Never	1.08 (1.06–1.10)	7.74	<0.001
Former	1.09 (1.07–1.09)	9.88	<0.001
Current	1.08 (1.06–1.11)	6.90	<0.001
**Alcohol**			
Never	1.07 (1.04–1.10)	5.00	<0.001
None in past year	1.10 (1.08–1.12)	10.30	<0.001
<1 drink per month	1.06 (1.03–1.09)	4.16	<0.001
1–3 drinks per month	1.08 (1.04–1.12)	3.79	<0.001
1–3 drinks per week	1.09 (1.06–1.14)	4.83	<0.001
4+ drinks per week	1.13 (1.10–1.17)	7.64	<0.001
Binge drinking*			
Yes	1.11 (1.06–1.15)	5.11	<0.001
No	1.09 (1.08–1.10)	14.43	<0.001
**Disease count**			
0	1.08 (1.05–1.12)	4.54	<0.001
1	1.08 (1.06–1.11)	6.66	<0.001
2	1.09 (1.05–1.13)	4.77	<0.001
3+	1.04 (1.01–1.08)	2.14	0.032
**BMI category**^†^			
Underweight	1.15 (1.04–1.27)	2.79	0.005
Normal	1.07 (1.05–1.10)	5.61	<0.001
Overweight	1.10 (1.07–1.12)	9.00	<0.001
Obese	1.10 (1.08–1.12)	10.57	<0.001
**Healthy**^‡^	1.08 (1.03–1.14)	2.94	0.003

Results are based on parametric survival models (Gompertz distribution). All models were adjusted for chronological age and sex.

*Binge drinking was defined at having 5+ alcoholic beverages at a time at least once per month.

^†^Underweight was defined as BMI < 18.5 kg/m^2^, normal was defined as 18.5 ≤ BMI < 25.0 kg/m^2^, overweight was defined as 25.0 ≤ BMI < 30.0 kg/m^2^, and obese was defined as BMI ≥ 30 kg/m^2^.

^‡^Healthy participants were defined as those having no disease and normal BMI.

BMI, body mass index; GED, general educational development; HS, high school.

Additionally, given the importance of identifying at-risk persons as early as possible, we evaluated whether Phenotypic Age was associated with all-cause mortality among persons who appeared clinically healthy (defined as having no disease and normal BMI). As shown in Table 3, among those healthy participants (*n* = 1,906), we observed that a 1-year increase in Phenotypic Age was still associated with an 8% increase in all-cause mortality risk.

### Association of Phenotypic Age with all-cause mortality in oldest-old adults

Table 4 provides the mortality association in oldest-old adults. We found that regardless of adjustment, Phenotypic Age was associated with mortality in this subpopulation, although to a lesser degree than in the full population (unadjusted model: HR = 1.05, 95% CI = 1.01–1.08; disease-adjusted model: HR = 1.05, 95% CI = 1.02–1.08).

**Table 4:** Association between Phenotypic Age and all-cause mortality in oldest-old adults (aged 85+ years).

Model	Hazard ratio (95% CI)	*z-*Score	*P* value
Without disease count adjustment	1.05 (1.01–1.08)	2.73	0.006
With disease count adjustment	1.05 (1.02–1.08)	3.21	0.001

Results are based on parametric survival models (Gompertz distribution). Models were not adjusted for chronological age (but adjusted for sex), given that this age group was top-coded at age 85 years in NHANES IV.

### Comparison to a previous clinical aging measure

Since another similar aging measure—Levine Biological Age (BioAge), which utilizes the Klemera and Doubal algorithm—currently provides one of the most accurate mortality predictors [13], we performed an additional analysis comparing the associations and predictions of Phenotypic Age to those of this measure. The results are provided in S1 Appendix, S2–S4 Tables, and S1 and S2 Figs. Overall, our results suggested that Phenotypic Age and Levine Biological Age were largely comparable, but Phenotypic Age performed better in the healthy subpopulation (e.g., those having no disease and normal BMI).

## Discussion

In a nationally representative US adult population, we showed that our new measure of aging—Phenotypic Age—was highly predictive of mortality even after adjusting for chronological age. Overall, we found that the mortality prediction of this measure is valid across different stratifications, particularly by age, disease count, health behaviors, and cause of death. For instance, Phenotypic Age is strongly associated with all-cause mortality in multiple age groups, including young adults, middle aged adults, and older adults. Moreover, the effect sizes seem to decrease with age, which may suggest that in younger groups, when the risk of death is low, variations in physiological status—as captured by PhenoAgeAccel—may play a bigger role in who lives longer. Conversely, in older adults, for whom the risk of death increases, mortality may be more stochastic. Nevertheless, we were able to determine that this measure was not just capturing an end-of-life or critically ill status, given that it remained predictive of mortality after excluding participants who had not survived for at least 5 years after baseline.

The finding that Phenotypic Age was predictive of mortality among both healthy and unhealthy populations even after adjusting for chronological age is novel. Many of the measures of aging, such as those based on deficit accumulation [14,27], include measures of morbidity in their construction, and thus it is impossible to disentangle aging and disease, or determine the usefulness of such measures in healthy populations. Belsky et al. evaluated aging measures, including Levine Biological Age, in a cohort study of young adults who were mostly disease-free [15,16]. However, the outcomes available were mostly restricted to functional assessments, which may mean something different in younger adults than they do in older populations. Conversely, in this study, we were able to show that Phenotypic Age was predictive of all-cause mortality among disease-free, healthy adults across the age spectrum. This suggests that Phenotypic Age is not simply a measure of disease or morbidity and instead may be a marker that tracks the effect of aging before diseases become clinically evident. This suggests that in a clinical setting, PhenoAgeAccel could be used to stratify risk among persons who otherwise “appear” healthy.

As expected of an aging biomarker, PhenoAgeAccel also tracks multimorbidity. We observed a strong association between the number of diseases a person reported being diagnosed with and his/her Phenotypic Age relative to his/her chronological age. Despite relatively small sample sizes, in general, PhenoAgeAccel appeared to increase as a function of disease count, suggesting that among persons of the same age, the more coexisting diseases a person has, the phenotypically older he/she appears—based on clinical biomarkers. Nevertheless, PhenoAgeAccel predicted risk of death significantly better than disease count, suggesting that it is capturing information beyond a person’s number of coexisting conditions. This is further supported by the significant association of PhenoAgeAccel with mortality in oldest-old adults—a population with high disease prevalence—and, more importantly, this association remained even after adjusting for disease count.

The efficacy of Phenotypic Age for assessing mortality risk in the general population, as well as multiple subpopulations that are heterogeneous in age and health status, provides strong evidence of its suitability for applications in both the clinical setting and research in the biology of aging. For instance, the generalizability of Phenotypic Age in assessing the risk of various aging outcomes may facilitate identification of at-risk individuals for a number of distinct conditions. Phenotypic Age may also be a useful marker for evaluation of interventions—particularly those concerned with prevention via delaying disease pathogenesis [18,28–30]. Aging changes are hypothesized to begin as early as conception [31]—preceding disease—thus interventions to slow aging will be most effective for reducing disease incidence if started early in the life course prior to significant accumulation of aging-related damage. Our findings suggest that Phenotypic Age is in line with the Geroscience paradigm, which stipulates that “aging is the greatest risk factor for a majority of chronic diseases driving both morbidity and mortality” [32,33]. Therefore, measures such as Phenotypic Age that capture pre-clinical aging as well as future morbidity/mortality risk could facilitate evaluation of intervention efficacy, while avoiding the need for decades of follow-up [28]. While research to develop interventions that target the aging process is ongoing, our paper provides a potential end point for which they can be evaluated. Further, this metric may also shed light on factors that alter the pace of aging, facilitating investigation into potential biological mechanisms and environmental stressors.

Despite the promising applications of Phenotypic Age, one limitation of this study is the lack of longitudinal data for either Phenotypic Age or disease incidence. As such, we were unable to confirm whether higher PhenoAgeAccel is predictive of disease accumulation (e.g., among persons with 1 disease, whether PhenoAgeAccel predicts who will develop a second comorbid condition). We were also unable to distinguish the mortality risks associated with (1) the rate of change in Phenotypic Age (true acceleration) versus (2) the baseline level of Phenotypic Age relative to chronological age.

In conclusion, our study shows that after adjusting for chronological age, Phenotypic Age, a novel clinically based measure of aging, is predictive of remaining life expectancy in a nationally representative population. Importantly, its prediction is robust to population characteristics—it is a reliable mortality predictor regardless of the age or health status of the population being assessed. Further, this measure captures both all-cause and disease-specific mortality, and is also strongly associated with the number of comorbid conditions. These findings suggest that this new aging measure may serve as a useful tool to facilitate identification of at-risk individuals and evaluation of intervention efficacy. Nevertheless, further evaluation in other cohorts is needed.

## Supporting information

S1 Appendix Additional analyses for Levine BioAge. (DOCX)

S1 Fig Predicted increases in Levine BioAge Acceleration (BioAgeAccel) for each disease count by age category. The *y-*axis depicts the increase in BioAgeAccel in comparison to persons who were disease-free. The *x-*axis shows groups categorized based on chronological age and the number of diseases each participant had.
(TIF)

S2 Fig Receiver operating characteristic curves of 10-year mortality. We evaluated the accuracy in predicting 10-year mortality for 3 variables—Phenotypic Age, Levine BioAge, and chronological age—within 2 samples: the full sample (A) and the healthy sample (defined as those having no disease and normal body mass index) (B). Persons who were not observed for at least 10 years were excluded. We found that for the full sample, Phenotypic Age was the best predictor of mortality. In the healthy sample, Phenotypic Age performed better, yet the differences in prediction were not significant. However, it should be pointed out that only 560 healthy participants were followed up for a minimum of 10 years. Therefore, these differences may become significant with larger samples. AUC, area under the curve; SE, standard error.
(TIF)

S1 STROBE STROBE checklist for the study. (DOC)

S1 Table Characteristics of the study participants, NHANES IV, 1999–2010. (DOCX)

S2 Table Associations of Levine BioAge with all-cause and disease-specific mortality. (DOCX)

S3 Table Associations between Levine BioAge and all-cause mortality in population subgroups. (DOCX)

S4 Table Associations between Levine BioAge and all-cause mortality in oldest-old adults (aged 85+ years). (DOCX)

## Abbreviations

AUC: area under the curve
BMI: body mass index
CRP: C-reactive protein
GED: general educational development
HR: hazard ratio
HS: high school
PhenoAgeAccel: Phenotypic Age Acceleration
ROC: receiver operating characteristic
SES: socioeconomic status
