# Correction: A new aging measure captures morbidity and mortality risk across diverse subpopulations from NHANES IV: A cohort study

**DOI:** 10.1371/journal.pmed.1002760

**Published:** 2019-02-25

**Authors:** Zuyun Liu, Pei-Lun Kuo, Steve Horvath, Eileen Crimmins, Luigi Ferrucci, Morgan Levine

The equation in the subsection of the Methods section titled “Phenotypic Age” is missing a step which makes it impossible to resolve. The equation should read:

$$Phenotypic\;Age=141.50+\frac{\ln\left[-0.00553\times \ln\left(1-M\right)\right]}{0.09165}$$

Where:

$$M=1-exp\left(\frac{-1.51714\times exp(xb)}{0.0076927}\right)$$

and:

$$\begin{array}{l} xb=-19.907-0.0336\times Albumin+0.0095\times Creatinine+0.1953\times Glucose\\ \;+0.0954\times \ln\left(CRP\right)-0.0120\times Lymphocyte\;Percent\\ \;+0.0268\times Mean\;Cell\;Volume+0.3306\times Red\;Cell\;Distribution\;Width\\ \;+0.00188\times Alkaline\;Phosphatase+0.0554\times White\;Blood\;Cell\;Count\\ \;+0.0804\times Chronological\;Age \end{array}$$
