# Supplementary material for: A new aging measure captures morbidity and mortality risk across diverse subpopulations from NHANES IV: A cohort study
Source: PLoS Med. 2018 Dec 31;15(12):e1002718. doi: 10.1371/journal.pmed.1002718 (PMC6312200; doi:10.1371/journal.pmed.1002718)
Supplement: S1 Appendix — (DOCX) [file pmed.1002718.s001.docx]

**S1 Appendix.** Additional analyses for Levine BioAge

We also evaluated the same models for our previous clinical composite aging measure, Levine Biological Age (BioAge), also commonly referred to as KDM-BA.

**Methods**

**Levine BioAge**

We calculated the Levine BioAge for each participant using methods outlined in [1] and [2]. The equation is based on a mathematical algorithm proposed by Klemera and Doubal [1], that takes information from m number of regression lines of chronological age regressed on m biomarkers (m=eight in this study). Previously published parameters [2] estimated from the NHANES III data were used for eight biomarkers (i.e., C-reactive protein, serum creatinine, glycated hemoglobin, serum albumin, serum total cholesterol, serum urea nitrogen, serum alkaline phosphatase, and systolic blood pressure).

As done for Phenotypic Age, we also calculated a measure of BioAge acceleration (BioAgeAccel), defined as the residual resulting from a linear model when regressing BioAge on chronological age. Therefore, BioAgeAccel represents biological aging after accounting for chronological age (i.e., whether a person appears older [positive value] or younger [negative value] than expected, physiologically).

**Results**

**BioAgeAccel according to disease counts and age categories**

As shown in S1 Fig, in young adults, BioAgeAccel was 0.17 years higher for those with one disease and 0.45 years higher for those with two diseases, compared to disease-free participants. In the middle aged group, BioAgeAccel was 0.19 years higher for those with one disease, 0.31 years higher for those with two diseases, and about half a year higher for those with three or more diseases, compared to those free of disease. Finally, older adults with one or two diseases were about 0.13 years older than older disease-free participants, those with three diseases were 0.34 years older, and those with four or more diseases were about 0.38 years older, according to BioAgeAccel.

**Associations with all-cause and disease-specific mortality**

S2 Table shows the associations of BioAge with all-cause and disease-specific mortality, based on proportional hazard models with Gompertz distribution. In the full sample, each one-year increase in BioAge (after adjusting for chronological age) increased the risk of mortality by 10% (Hazard ratio [HR]=1.10, 95% confidence interval [CI]=1.08-1.13). When re-evaluating mortality associations after restricting the sample to participants who survived at least five years after baseline, we found consistent results (HR=1.09, 95%CI=1.04-1.14). When examining mortality within age stratified groups, we found that BioAge was predictive in all groups, such that each one-year increase in BioAge increased mortality by 12% in young adults (HR=1.12, 95%CI=1.07-1.18), 13% in middle aged adults (HR=1.13, 95%CI=1.10-1.17), and 7% in older adults (HR=1.07, 95%CI=1.04-1.10).

When examining disease-specific mortality using competing risk hazard models, we found that BioAge was associated with heart disease mortality (HR=1.14, 95%CI=1.10-1.17), cerebrovascular disease (HR=1.08, 95%CI=1.00-1.15), diabetes mortality (HR=1.20, 95%CI=1.15-1.24), influenza/pneumonia mortality (HR=1.10, 95%CI=1.02-1.20), and nephritis, nephrotic syndrome, and nephrosis mortality (HR=1.22, 95%CI=1.17-1.28). However, BioAge was not associated with cancer mortality (HR=1.00, 95%CI=0.96-1.04) nor chronic lower respiratory-specific mortality (HR=0.92, 95%CI=0.79-1.07).

**Associations with all-cause mortality in population subgroups**

As shown in S3 Table, we observed nonsignificant association of BioAge with all-cause mortality in participants with no disease (HR=1.00, 95%CI=0.92-1.08). Among those having no disease and normal BMI (defined as healthy), we found that after adjusting for chronological age and sex, BioAge did not predict all-cause mortality (HR=0.94, 95%CI=0.84-1.05).

The prediction of BioAge for all-cause mortality was comparable by race/ethnicity, education (except the college subgroup), and smoking. For example, in the non-Hispanic white, the non-Hispanic black, and the Hispanic, a one-year increase in BioAge was associated with all-cause mortality, with HR ranging from 1.09 to 1.12. BioAge did not predict all-cause mortality among those who drank <1 drink per month, 1-3 drinks per week, or 4+ drinks per week. We also found a nonsignificant result among binge drinkers. When all variables were adjusted for, BioAge was significantly association with mortality (HR=1.05, P=0.003).

**Associations with all-cause mortality in the oldest-old**

S4 Table provides the mortality associations for BioAge in the oldest-old. We found that in models without and with disease count adjustment, BioAge was significantly associated with all-cause mortality (HR=1.04, 95%CI=1.01-1.07 for both models).

S2 Fig compared the accuracy in predicting ten-year mortality for three variables—Phenotypic Age, BioAge, and chronological age in full sample (S2 Fig A) and healthy samples (S2 Fig B). We found that in the full sample, Phenotypic Age was the best predictor of mortality. In the healthy sample, Phenotypic Age performed better, yet the differences in prediction were not significant—this may be due to the reduction in sample size when limiting sample to those with no disease and BMI in the normal range.

**References**

1. Klemera P, Doubal S. A new approach to the concept and computation of biological age. Mech Ageing Dev. 2006;127(3):240-8. doi: 10.1016/j.mad.2005.10.004. PMID: 16318865.

2. Levine ME. Modeling the rate of senescence: can estimated biological age predict mortality more accurately than chronological age? J Gerontol A Biol Sci Med Sci. 2013;68(6):667-74. doi: 10.1093/gerona/gls233. PMID: 23213031.
