# Supplementary figures and images for: A new aging measure captures morbidity and mortality risk across diverse subpopulations from NHANES IV: A cohort study
Source: PLoS Med. 2018 Dec 31;15(12):e1002718. doi: 10.1371/journal.pmed.1002718 (PMC6312200; doi:10.1371/journal.pmed.1002718)

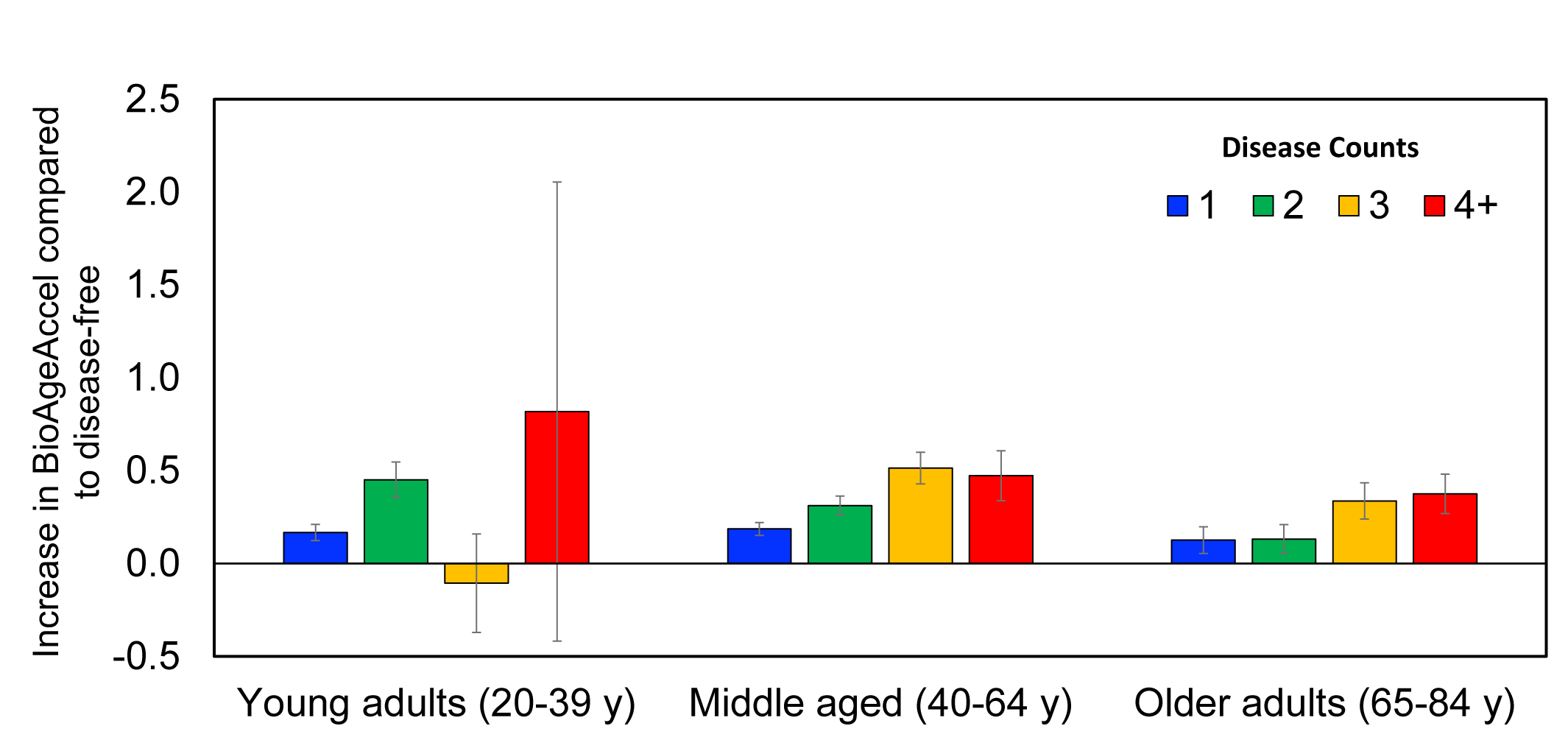

Supplement: S1 Fig — The y-axis depicts the increase in BioAgeAccel in comparison to persons who were disease-free. The x-axis shows groups categorized based on chronological age and the number of diseases each participant had. (TIF) [file pmed.1002718.s002.tif]

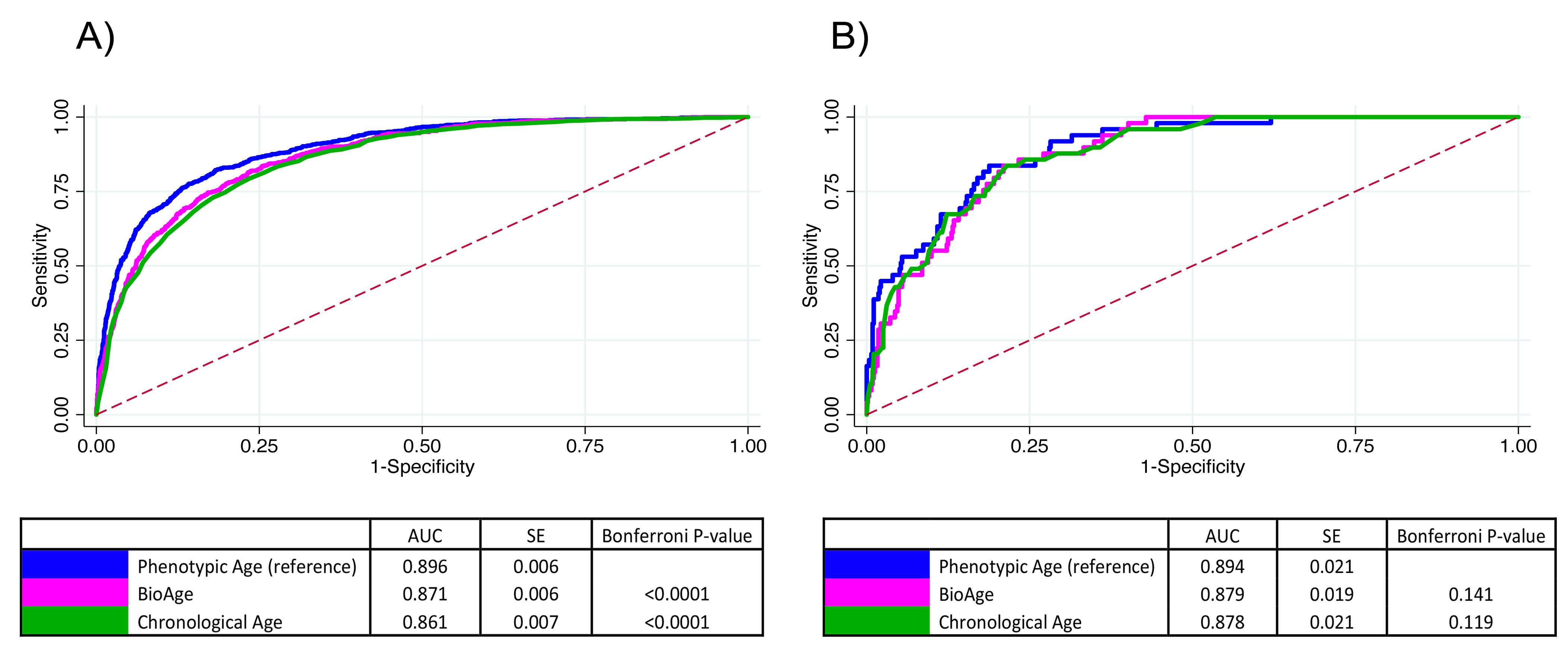

Supplement: S2 Fig — We evaluated the accuracy in predicting 10-year mortality for 3 variables—Phenotypic Age, Levine BioAge, and chronological age—within 2 samples: the full sample (A) and the healthy sample (defined as those having no disease and normal body mass index) (B). Persons who were not observed for at least 10 years were excluded. We found that for the full sample, Phenotypic Age was the best predictor of mortality. In the healthy sample, Phenotypic Age performed better, yet the differences in prediction were not significant. However, it should be pointed out that only 560 healthy participants were followed up for a minimum of 10 years. Therefore, these differences may become significant with larger samples. AUC, area under the curve; SE, standard error. (TIF) [file pmed.1002718.s003.tif]
