# Supplementary material for: A new aging measure captures morbidity and mortality risk across diverse subpopulations from NHANES IV: A cohort study
Source: PLoS Med. 2018 Dec 31;15(12):e1002718. doi: 10.1371/journal.pmed.1002718 (PMC6312200; doi:10.1371/journal.pmed.1002718)
Supplement: S1 Table — (DOCX) [file pmed.1002718.s005.docx]

**S1 Table. Characteristics of the study participants, NHANES IV, 1999-2010***

| **Characteristics** | **No. (%) or mean ± SD** |
| --- | --- |
| All | 11432 |
| Age, y | 45.5 ± 18.6 |
| Young adults (20-39 y) | 4,016 (40.0) |
| Middle aged (40-64 y) | 4,784 (45.2) |
| Older adults (65-84 y) | 2,632 (14.8) |
| Female | 5,856 (50.8) |
| Race/ethnicity† |  |
| Non-Hispanic white | 5,709 (75.6) |
| Non-Hispanic black | 2,023 (10.9) |
| Hispanic | 3,251 (13.4) |
| Education |  |
| <HS | 3,358 (18.8) |
| HS/GED | 2,710 (25.4) |
| Some college | 3,104 (30.2) |
| College | 2,245 (25.6) |
| Smoking |  |
| Never | 5,973 (51.3) |
| Former | 2,993 (25.3) |
| Current | 2,456 (23.4) |
| Alcohol |  |
| Never | 1,486 (11.1) |
| None in past year | 2,164 (16.5) |
| <1 drink per month | 2,145 (19.1) |
| 1-3 drinks per month | 1,527 (15.2) |
| 1-3 drinks per week | 2,051 (21.6) |
| 4+ drinks per week | 1,542 (16.7) |
| Binge drinking |  |
| Yes | 1,488 (14.5) |
| No | 9,944 (85.5) |
| BMI categories |  |
| Underweight | 165 (1.8) |
| Normal | 3,243 (32.1) |
| Overweight | 4,016 (33.9) |
| Obese | 3,881 (32.2) |
| Disease counts |  |
| 0 | 5,768 (66.5) |
| 1 | 1,978 (21.1) |
| 2 | 776 (8.0) |
| 3 | 286 (2.8) |
| 4+ | 167 (1.6) |
| Healthy‡ | 1,906 (19.7) |
| Died | 871 (5.3) |
| 5+ years survival | 389 (2.5) |
|  |  |
| Biomarkers selected for calculating Phenotypic Age | |
| Albumin, mg/dL | 42.7 ± 4.2 |
| Creatinine, µmol/L | 75.2 ± 21.9 |
| Glucose, mmol/L | 5.4 ± 1.5 |
| C-reactive protein, g/L | 0.4 ± 1.0 |
| Lymphocyte percent, % | 30.0 ± 9.6 |
| Mean cell volume, fL | 90.0 ± 6.0 |
| Red cell distribution width, % | 12.7 ± 1.1 |
| Alkaline phosphatase, U/L | 69.4 ± 28.0 |
| White blood cell count, 1000cell/uL | 6.8 ± 2.4 |

SD, standard deviation; HS, high school; GED, general educational development; BMI, body mass index. No. was based on study participants (unweighted). Percentages were weighted estimates and may not sum to 100 because of rounding. There were missing data on education (n=15), smoking (n=10), BMI categories (n=127), and disease (n=2,457).

* The oldest-old (aged 85+, n =185) were not considered here, as described in the Methods.

† 449 participants who self- identified as other race (including multi-racial) were not considered.

‡ Healthy participants were defined as those having no disease and normal BMI.
