# Supplementary material for: A new aging measure captures morbidity and mortality risk across diverse subpopulations from NHANES IV: A cohort study
Source: PLoS Med. 2018 Dec 31;15(12):e1002718. doi: 10.1371/journal.pmed.1002718 (PMC6312200; doi:10.1371/journal.pmed.1002718)
Supplement: S2 Table — (DOCX) [file pmed.1002718.s006.docx]

**S2 Table.** **Associations of Levine BioAge with all-cause and disease-specific mortality**

|  |  | No. of death | Hazard Ratio  (95% CI) | z-score | P  value |
| --- | --- | --- | --- | --- | --- |
| All-cause | Full sample | 871 | 1.10 (1.08-1.13) | 9.05 | <0.001 |
|  | All-cause (5+ years survival) | 389 | 1.09 (1.04-1.14) | 3.68 | <0.001 |
|  | Young adults (20-39 y) | 32 | 1.12 (1.07-1.18) | 4.41 | <0.001 |
|  | Middle aged (40-64 y) | 247 | 1.13 (1.10-1.17) | 8.26 | <0.001 |
|  | Older adults (65-84 y) | 592 | 1.07 (1.04-1.10) | 4.92 | <0.001 |
| Disease-specific | Heart disease | 141 | 1.14 (1.10-1.17) | 7.37 | <0.001 |
|  | Cancer | 227 | 1.00 (0.96-1.04) | -0.03 | 0.976 |
|  | Chronic lower respiratory disease | 52 | 0.92 (0.79-1.07) | -1.09 | 0.276 |
|  | Cerebrovascular disease | 56 | 1.08 (1.00-1.15) | 2.07 | 0.038 |
|  | Diabetes | 26 | 1.20 (1.15-1.24) | 9.23 | <0.001 |
|  | Influenza or pneumonia | 24 | 1.10 (1.02-1.20) | 2.35 | 0.019 |
|  | Nephritis/nephrosis | 15 | 1.22 (1.17-1.28) | 8.49 | <0.001 |

CI, confidence interval. Results are based on Parametric Survival Models (Gompertz distribution). All models were adjusted for chronological age and sex.
