# Supplementary material for: A new aging measure captures morbidity and mortality risk across diverse subpopulations from NHANES IV: A cohort study
Source: PLoS Med. 2018 Dec 31;15(12):e1002718. doi: 10.1371/journal.pmed.1002718 (PMC6312200; doi:10.1371/journal.pmed.1002718)
Supplement: S3 Table — (DOCX) [file pmed.1002718.s007.docx]

**S3 Table. Associations between Levine BioAge and all-cause mortality in population subgroups**

|  | |  | Hazard Ratio  (95% CI) | z-score | P value |
| --- | --- | --- | --- | --- | --- |
| Race/ethnicity | | |  |  |  |
|  | Non-Hispanic white | | 1.10 (1.07-1.14) | 6.01 | <0.001 |
|  | Non-Hispanic black | | 1.09 (1.06-1.12) | 5.81 | <0.001 |
|  | Hispanic | | 1.12 (1.08-1.16) | 5.81 | <0.001 |
| Education | | |  |  |  |
|  | <HS | | 1.09 (1.05-1.12) | 5.36 | <0.001 |
|  | HS/GED | | 1.10 (1.05-1.15) | 4.28 | <0.001 |
|  | Some college | | 1.11 (1.06-1.16) | 4.78 | <0.001 |
|  | College | | 1.06 (0.97-1.17) | 1.30 | 0.194 |
| Smoking | | |  |  |  |
|  | Never | | 1.11 (1.08-1.15) | 6.25 | <0.001 |
|  | Former | | 1.09 (1.05-1.13) | 4.57 | <0.001 |
|  | Current | | 1.09 (1.05-1.13) | 4.80 | <0.001 |
| Alcohol | | |  |  |  |
|  | Never | | 1.09 (1.05-1.14) | 4.01 | <0.001 |
|  | None in past year | | 1.11 (1.08-1.14) | 6.94 | <0.001 |
|  | <1 drink per month | | 1.03 (0.94-1.12) | 0.62 | 0.537 |
|  | 1-3 drinks per month | | 1.11 (1.04-1.19) | 2.94 | 0.003 |
|  | 1-3 drinks per week | | 1.07 (1.00-1.15) | 1.94 | 0.053 |
|  | 4+ drinks per week | | 1.10 (1.00-1.20) | 2.00 | 0.046 |
| Binge drinking* | | |  |  |  |
|  | Yes | | 1.05 (0.96-1.14) | 0.99 | 0.321 |
|  | No | | 1.11 (1.08-1.13) | 9.10 | <0.001 |
| Disease Count | | |  |  |  |
|  | 0 | | 1.00 (0.92-1.08) | -0.06 | 0.956 |
|  | 1 | | 1.07 (1.02-1.13) | 2.61 | 0.009 |
|  | 2 | | 1.12 (1.07-1.19) | 4.36 | <0.001 |
|  | 3+ | | 1.08 (1.01-1.15) | 2.34 | 0.019 |
| BMI Categories† | | |  |  |  |
|  | Underweight | | 0.96 (0.82-1.13) | -0.45 | 0.650 |
|  | Normal | | 1.06 (1.02-1.11) | 2.74 | 0.006 |
|  | Overweight | | 1.09 (1.04-1.14) | 3.76 | <0.001 |
|  | Obese | | 1.13 (1.10-1.17) | 9.01 | <0.001 |
| Healthy‡ | | | 0.94 (0.84-1.05) | -1.14 | 0.256 |

CI, confidence interval; HS, high school; GED, general educational development; BMI, body mass index. Results are based on Parametric Survival Models (Gompertz distribution). All models were adjusted for chronological age and sex.

*Binge drinking was defined at having 5+ alcoholic beverages at a time at least once per month. †Underweight was defined as BMI<18.5; normal was defined as a value between 18.5 and 25.0; overweight was defined as a value between 25.0 and 30.0; and obese was defined as BMI of 30 or greater.

‡Healthy participants were defined as those having no disease and normal BMI.
