# Supplementary material for: A new aging measure captures morbidity and mortality risk across diverse subpopulations from NHANES IV: A cohort study
Source: PLoS Med. 2018 Dec 31;15(12):e1002718. doi: 10.1371/journal.pmed.1002718 (PMC6312200; doi:10.1371/journal.pmed.1002718)
Supplement: S4 Table — (DOCX) [file pmed.1002718.s008.docx]

**S4 Table. Associations between Levine BioAge and all-cause mortality in the oldest-old (aged 85+)**

|  | Hazard Ratio  (95% CI) | Z  score | P  value |
| --- | --- | --- | --- |
| Model 1: without disease count adjustment | 1.04 (1.01-1.07) | 2.63 | 0.009 |
| Model 2: with disease count adjustment | 1.04 (1.01-1.07) | 2.72 | 0.007 |

CI, confidence interval. Results are based on Parametric Survival Models (Gompertz distribution). Models were not adjusted for chronological age (but adjusted for sex), given that it was top-coded at age 85 in NHANES IV.
